# Association of *IL-4* and *IL-10* maternal haplotypes with immune responses to *P. falciparum* in mothers and newborns

**DOI:** 10.1186/1471-2334-13-215

**Published:** 2013-05-13

**Authors:** Adjimon Gatien Lokossou, Célia Dechavanne, Aziz Bouraïma, David Courtin, Agnès Le Port, Rodolphe Ladékpo, Julien Noukpo, Désiré Bonou, Claude Ahouangninou, Audrey Sabbagh, Benjamin Fayomi, Achille Massougbodji, André Garcia, Florence Migot-Nabias

**Affiliations:** 1 Institut de Recherche pour le Développement, UMR 216 Mère et enfant face aux infections tropicales, Paris, France; 2 PRES Sorbonne Paris Cité, Université Paris Descartes, Faculté de Pharmacie, Paris, France; 3 Centre d’Étude et de Recherche sur le Paludisme Associé à la Grossesse et l’Enfance (CERPAGE), Cotonou, Bénin; 4 Institut des Sciences Biomédicales Appliquées, Cotonou, Bénin; 5 Laboratoire de Parasitologie, Faculté des Sciences de la Santé, Cotonou, Bénin; 6 Present address: CRCHUM-Hôpital Saint-Luc, 264 boulevard René-Lévesque Est, Montreal (Quebec) H2X 1P1, Canada

**Keywords:** Malaria, *P. falciparum*, Cytokine gene polymorphisms, IL-4, IL-10, IL-13, Pregnancy, Cord blood, Recombinant proteins, Specific antibodies

## Abstract

**Background:**

Particular cytokine gene polymorphisms are involved in the regulation of the antibody production. The consequences of already described *IL-4*, *IL-10* and *IL-13* gene polymorphisms on biological parameters and antibody levels were investigated among 576 mothers at delivery and their newborns in the context of *P. falciparum* placental malaria infection.

**Methods:**

The study took place in the semi-rural area of Tori-Bossito, in south-west Benin, where malaria is meso-endemic. Six biallelic polymorphisms were determined by quantitative PCR using TaqMan® Pre-Designed SNP Genotyping Assays, in *IL-4* (rs2243250, rs2070874), *IL-10* (rs1800896, rs1800871, rs1800872) and *IL-13* (rs1800925) genes. Antibody responses directed to *P. falciparum* MSP-1, MSP-2, MSP-3, GLURP-R0, GLURP-R2 and AMA-1 recombinant proteins were determined by ELISA.

**Results:**

The maternal *IL-4*_−590_*T/IL-4_+33_*T haplotype (one or two copies) was associated with favorable maternal condition at delivery (high haemoglobin levels, absence of placental parasites) and one of its component, the *IL-4*_*−590*_*TT* genotype, was related to low IgG levels to MSP-1, MSP-2/3D7 and MSP-2/FC27. Inversely, the maternal *IL-10*_*−1082*_*AA* was positively associated with *P. falciparum* placenta infection at delivery. As a consequence, the *IL-10*_−819_*T allele (in *CT* and *TT* genotypes) as well as the *IL-10*_−1082_*A/IL-10_−819_*T/IL-10_−592_*A haplotype (one or two copies) in which it is included, were related to an increased risk for anaemia in newborns. The maternal *IL-10*_*−1082*_*AA* genotype was related to high IgG levels to MSP-2/3D7 and AMA-1 in mothers and newborns, respectively. The *IL-13* gene polymorphism was only involved in the newborn’s antibody response to AMA-1.

**Conclusion:**

These data revealed that *IL-4* and *IL-10* maternal gene polymorphisms are likely to play a role in the regulation of biological parameters in pregnant women at delivery (anaemia, *P. falciparum* placenta infection) and in newborns (anaemia). Moreover, *IL-4*, *IL-10* and *IL-13* maternal gene polymorphisms were related to IgG responses to MSP-1, MSP-2/3D7 and MSP-2/FC27 in mothers as well as to AMA-1 in newborns.

## Background

Host susceptibility to malaria is attributable to a number of factors, which include the genetic background of both host and pathogen. Indeed host genetic factors are involved in the regulation of the individual’s immunological competence. Several chromosomal regions containing genes coding for cytokines or cytokine receptors have been implicated in the control of *P. falciparum* infection levels [1-3]. The 5q31-q33 region located in chromosome 5 notably contains a number of genes initially found associated with the immune response directed to *Schistosoma haematobium*[4]. In the case of malaria, studies have provided evidence for linkage between the 5q31-q33 region and blood infection levels in populations originating from Burkina Faso [5], Cameroon [6] and Senegal [7]. Interleukin (IL) genes such as *IL-3*, *IL-4*, *IL-5*, *IL-9* and *IL-13* are notably clustered in this chromosome region [5,8].

The products of the pleiotropic *IL-4* gene intervene in multiple immune modulating functions depending on a variety of cell types [9]. IL-4 is defined as a cytokine produced by Th2 cells, and is involved in the regulation of the humoral immune response. It is a key factor for the differentiation of precursor T helper cells into Th2 cells that induce IgE production by plasmocytes. This cytokine is an important regulator in the isotype switching from IgM/IgG to IgE [10]. Finally, IL-4 plays a critical role in the regulation of the antibody response induced by *Plasmodium* parasites [8,11]. A gene located in the 1q32.1 region of chromosome 1 encodes IL-10 which synergizes the production of antibody isotypes (IgG, IgA and IgM) induced by IL-4 [12]. IL-4 and IL-10 have been shown to be important for parasite clearance in later antibody-mediated phases of infection [13]. Among the genes located in the 5q31.1 region, *IL-13* encodes a cytokine which is a central mediator of the physiological changes induced by allergic inflammation. The functions of IL-13 considerably overlap those of IL-4, especially with regard to their role on erythropoiesis. IL-13 has anti-inflammatory properties and induces IgE secretion from activated human B cells [14].

Several polymorphisms affecting *IL-4*, *IL-10*, *IL-13* genes, lead to changes in cytokine production levels that may impact isotype switching as well as cell interaction and thus be associated with immune-related diseases such as malaria [15,16]. A variant at position *IL-4*_*−590*_*C/T* has been shown to enhance IL-4 and IgE production [17]. Studies in Burkina Faso [18] and Mali [19,20] revealed differences in the distribution of *IL-4*_*−590*_*C/T* allele and genotype frequencies between Fulani and non Fulani ethnic groups. In both studies, the *IL-4*_−590_*T allele was associated with high levels of anti-malaria IgG levels among Fulani. A study conducted among Ghanaian children showed that carriers of both *IL-4*_−590_*T and *IL-4*_+33_*T alleles presenting cerebral malaria had elevated IgE compared to non carriers [21].

There is evidence for associations between *IL-10* gene polymorphisms grouped into haplotypes and IL-10 levels as well as antibody levels. The *IL-10*_*−1082*_*G/A* polymorphism has notably been associated with variable IL-10 production [22]. The *IL-10*_−592_*A allele has been linked to low levels of total IgE and the *IL-10*_−1082_*A/IL-10_−592_*A haplotype has been negatively associated with specific IgE and IgG4 [23]. *IL-10*_−819_*C was associated with skin lesions induced by leishmaniasis, in relation with high IL-10 levels produced by carriers of the *IL-10*_*−819*_*CC* genotype [24]. Besides, low IL-10 producing *IL-10*_−1082_*A/IL-10_−819_*T/IL-10_−592_*A haplotype carriers may be at risk of severe malarial anaemia, among pediatric populations [25]. Finally, the *IL-13*_−1055_*T allele is found in individuals who present enhanced IL-13 production and seem protected against severe malaria [16].

In endemic areas, children aged less than 5 years are particularly affected by malaria because of their lack of specific acquired immunity. Several authors have reported that the susceptibility of the child aged less than one year is linked to malaria infections during pregnancy. Thus children born from mothers with a *P. falciparum* infected placenta at delivery have an increased risk of developing malaria during their first years of life [26-28]. Despite this information, knowledge on the genetic and immunological mechanisms related to increased susceptibility of pregnant women and their children to malaria is still lacking. We hypothesized that if specific maternal cytokine gene polymorphisms lead to an increased production of specific antimalarial antibodies at the individual level, they may also help to lower the *in utero* sensitization of the fetus to plasmodial antigens, and therefore contribute to delay the appearance of the first malaria attacks during the first months of life.

Studies conducted in different malaria endemic areas have reported that IgG to various malaria antigens were associated with clinical protection [29-31]. Passive antibody transfer to non-immune individuals showed that protective immunity among adults in endemic areas is at least partly humoral [32]. Cytophilic antibodies to merozoite surface protein-3 (MSP-3) and glutamate-rich protein (GLURP) were shown to be predominant in protected individuals, contrary to non-cytophilic antibodies [33]. Studies have also shown that antibodies directed to MSP-1_−19_ and apical membrane antigen 1 (AMA-1) were associated with a reduced risk of clinical malaria [34]. Antibodies to AMA-1 have been reported to exhibit parasite growth inhibitory activity in a growth inhibition assay [35]. Antibodies to merozoite surface protein-2 (MSP-2) of *P. falciparum* have been associated with protection from clinical malaria in independent studies [36]. Each of these antigens (AMA-1, MSP-1_19_, MSP-2, MSP-3, and GLURP) is considered as a candidate for inclusion into a multivalent malaria vaccine.

The aim of the present study was to verify the consequences of known cytokine gene polymorphisms on maternal biological parameters and specific antibody levels at delivery and to investigate their role on fetal health and immunity, taking into account the presence or absence of placental infection by *P. falciparum*. The simultaneous measurement of the antibody response directed against a panel of plasmodial antigens will allow drawing a picture of the specific immune status at the individual level.

## Methods

### Study area

The study was conducted in the rural area of Tori-Bossito, (45,000 inhabitants, 40 km west of Cotonou, Benin). Malaria transmission is perennial, with two seasonal peaks corresponding to rainy seasons, from April to July and September to November. *P. falciparum* is the main cause of infection and an average entomological inoculation rate (EIR) of 15.5 infective bites per person per year has been measured [37].

### Study population and sampling

A cohort of 660 mothers and their children was constituted from June 2007 to June 2008 in three maternities from the Tori-Bossito area, welcoming parturient women from nine surrounding villages [37]. In the present study, 576 mothers and their newborns were investigated, excluding twins and children who died before D7. At delivery, peripheral blood was drawn from all mothers and cords in EDTA Vacutainer® tubes (Becton Dickinson, Meylan, France). Plasma cytokine levels were measured from blood collected for 80 mothers and cords in supplementary heparinized Vacutainer® tubes. After collection, plasma samples were stored at −80°C and buffy-coats at −20°C.

Thick blood smears were prepared from circulating blood of mothers, cord blood and placenta samples. Staining with Giemsa allowed determining *P. falciparum* parasitaemia by microscopy. Leukocytes and parasites were counted simultaneously until leukocyte or parasite numbers reached 500. A thick blood smear was declared negative if no parasite was found after 500 leucocytes had been counted. The following variables were measured for each mother: (1) village of residence; (2) age; (3) parity and (4) estimated exposure to malaria transmission during pregnancy assessed by means of mosquito catches precisely described in Cottrell et al. [38]; (5) circulating parasite density and (6) presence of placental parasites at delivery.

Biological assays were assessed in part in the laboratory established at Cotonou by the UMR 216 in partnership with the Faculté des Sciences de la Santé (FSS) and the Institut des Sciences Biomédicales Appliquées (ISBA) in Benin, and in the laboratory of the UMR 216 in Paris, France. For each included parturient woman, a written informed consent was obtained. Both the Ethical Committee of the FSS in Benin and the Consultative Committee on Professional Conduct and Ethics (CCDE) of the Institut de Recherche pour le Développement (IRD) approved the protocol.

### Human genotyping

Genomic DNA was extracted from maternal buffy-coats containing mononuclear cells, using Qiamp® DNA blood midi kits according to the manufacturer’s instructions. Six cytokine gene polymorphisms were determined by quantitative PCR using TaqMan® Pre-Designed SNP (Single Nucleotide Polymorphism) Genotyping Assays, including the *C/T* mutation located at the −590 (rs2243250,) and +33 (rs2070874) positions of the *IL-4* gene, the *G/A* mutation at the −1082 (rs1800896), the *C/T* mutation at the −819 (rs1800871) and the *C/A* mutation at the −592 (rs1800872) positions of the *IL-10* gene, and the *C/T* mutation at the −1055 position (rs1800925) of the *IL-13* gene. Each TaqMan genotyping assay contained two primers for amplifying the sequence of interest and two TaqMan® MGB probes for detecting alleles. The presence of two probe pairs in each reaction allowed genotyping of the two possible variant alleles at the SNP site in the DNA target sequence. The genotyping assay determined the presence or absence of a SNP based on the change in fluorescence of the dyes associated with the probes. Amplification of each locus was performed in a total volume of 10 μl, containing 0.25 μM of each primer, 2 μl genomic DNA and 2× PCR Master Mix (Applied Biosystems master mix contains AmpliTaq Gold® DNA Polymerase, Ultra Pure water, deoxyribonucleotide triphosphates (dNTPs), ROX™ Passive Reference and Buffer components) in a programmable thermocycler (Applied Biosystems PRISM 7900HT®).

### Malaria antigens

Seven recombinant proteins corresponding to antigens of *P. falciparum* expressed on the surface of merozoites and candidates for inclusion in a multivalent asexual stage vaccine [39] were chosen. The recombinant proteins were obtained free of charge through an international network of collaborators. MSP-1_19_ (Uganda-Palo-Alto strain) (gift from Pasteur Institute, Paris, France) was expressed in a Baculovirus/insect cell system [40]; AMA-1 _25–545_ (FVO strain) (gift from the Biomedical Primate Research Centre, Rijswijk, The Netherlands) was expressed in *Pichia pastoris*[41]; MSP-2/3D7 and MSP-2/FC27 (gift from La Trobe University, Melbourne, Australia) [42] as well as MSP-3 _212–380_ (F32 strain) [43], GLURP _25–514_ (F32 strain) and GLURP _706–1178_ (F32 strain) [44] (all from the Statens Serum Institute, Copenhagen, Denmark) were expressed in *E. coli*. GLURP-R0 (amino acids 25–514) and -R2 (amino acids 706–1178) corresponded to the N-terminal nonrepeat region and to the C-terminal repeat region of the protein, respectively [30].

### Antibody measurements

An Enzyme-Linked Immuno-Sorbent Assay (ELISA) following a standardized methodology described in the Afro-immunoassay network standard operating procedure (procedures AIA-001-01 and −02) was used to assess the antibody response directed to the panel of *P. falciparum* recombinant proteins.

ELISA plates were coated overnight with 100 μl of recombinant protein solutions at a final concentration of 1 μg/ml in 1X PBS. Blocking buffer (3% milk powder in PBS - 0.1% Tween 20) was added (150 μl per well) and plates were kept at room temperature for 1 hour. Plasma samples were diluted in 1X PBS - 1% milk powder - 0.1% Tween 20-0.02% sodium azide. For IgG measurements, maternal and cord plasma samples were diluted 1:200 for all recombinant proteins except for AMA-1 where a dilution 1:2000 was used. For IgM measurements, plasma samples from maternal blood were diluted 1:200 and those of cord blood 1:50.

Two polyclonal antibodies conjugated to HRP were used: a goat anti-human IgG (Caltag H10007) and a goat anti-human IgM (Caltag H15007) both diluted 1: 3000. Bound enzyme was detected with TMB and the reaction was stopped with 0.2 M H_2_SO_4_ (100 μl/well). Plates were extensively washed between each incubation period with 1X PBS - 0.1% Tween 20-0.5 M NaCl. The optical density (OD) was read at 450 nm (reference filter 620 nm). Positive-control plasma samples from Gabonese individuals and negative-control plasma samples from Dutch individuals were included in each plate. A Microsoft Excel based curve fitting program (ADAMSEL FPL b039) containing a collection of macros and worksheets was used for calculating antibody concentrations (μg/ml) from the OD values, using the standard curves obtained from each ELISA test plate (http://www.emvda.bio.ed.ac.uk/software.php).

### Cytokine assays

IL-4, IL-10 and IL-13 plasma levels were measured in a sub-sample of 80 mothers presenting no difference in any criterion compared to the whole group of 576 mothers. Four of them presented *P. falciparum* parasites both in circulating blood and placenta. The DuoSet® ELISA tests (R&D systems) were used and results were expressed in pg/ml by reference to standard curves prepared in each plate with recombinant cytokines. Thresholds of sensitivity were 31 pg/ml for IL-4 and IL-10, and 94 pg/ml for IL-13.

### Statistical analysis

The significance of deviation from Hardy-Weinberg equilibrium was tested using the χ^2^ goodness-of-fit test. Strength of linkage disequilibrium (LD) between pairs of markers located along a same chromosome was measured as *r*^2^[45], using the Haploview software v4.1 [46]. Regions of strongly associated markers (LD blocks) were inferred using the confidence-interval method proposed by Gabriel et al. [47]. For SNPs located within a same LD block, haplotypes were inferred from the unphased genotype data using the Bayesian method implemented in PHASE v.2.1.1 [48], using default parameters. To avoid the convergence of the algorithm to a local maximum, we ran it 10 times with different random seeds and kept the output from the run with the best average value. The most probable haplotype constitution of each sample was also inferred with a maximum likelihood method using the Expectation-Maximization (EM) algorithm implemented in Arlequin v3.1 [49]. All samples had the same pair of haplotypes estimated by the EM and PHASE methods, therefore ensuring the reliability of the inference procedure.

For univariate analyses, differences in proportions were analyzed using the Chi square test. Differences in means were tested by the nonparametric Mann–Whitney *U*-test or Kruskal-Wallis test (for more than 2 groups to be compared). Statview 5.0 (SAS Institute Inc., Cary, NC) was used for these calculations. Results of the univariate analyses involving maternal cytokine gene polymorphisms are reported in the Additional files 1 and 2 (for biological parameters of either the mothers or the newborns) and in the Additional files 3 and 4 (for *P. falciparum* specific antibody levels of either the mothers or the newborns). Variables with *P* values < 0.20, were further considered in the multivariate analysis.

Multivariate analysis of the association between anaemia, placental infection and covariates was performed using multiple logistic regressions. For haemoglobin level, multivariate analyses were conducted using multiple linear regressions. Concerning antibody levels, due to non Gaussian distribution (even when data were log-transformed), median regression models were used. These models estimate standard errors via bootstrapping. We specified 1,000 replications for each analysis, and when significant (*P* < 0.05) a new analysis under the same model was carried out using 5,000 replications. Associations between biological and immunological phenotypes and each cytokine gene polymorphism were tested under additive, dominant and recessive genetic models.

The four tables reporting results of association, listed only cytokine gene polymorphisms for which multivariate analysis concluded to significant differences in relation to mothers and/or newborns biological and/or immunological parameters. For all tests, *P* values of less than 0.05 were considered significant. All analyses were performed with Stata software, version 11.0 (StatCorp LP, College Station, TX, USA).

### Results

#### Study population, clinical and parasitological characteristics

Haematological and parasitological data of the 576 included mothers and their newborns are presented in Table 1. Mothers distributed into 88 primigravidae (15%, mean age = 25.0 years) and 488 multigravidae (85%, mean age = 27.8 years). Primigravidae were more numerous than multigravidae to present circulating parasites at delivery (15 (17.0%) vs. 34 (7.0%), *P* = 0.002) but parasite densities did not differ (median parasite density/μl (IQR): 27 (5–514) vs. 5 (2–77), *P* = 0.08). Among the 563 mothers for whom placenta blood smears were available, 63 (10.9%) mothers presented a *P. falciparum* infected placenta at delivery. More positive smears were identified in primigravidae compared to multigravidae (17 (19.8%) vs. 46 (9.6%), *P* = 0.006). Anaemia was frequent, with 39.1% and 61.5% of mothers and newborns presenting haemoglobin values < 11 g/dl and < 15 g/dl, respectively. The mean birth weight (± SD) was 2985 g (± 388 g) with 53 newborns (9.2%) presenting a low birth weight (< 2500 g). The 5 newborns presenting parasites in cord blood were born of mothers with both circulating *P. falciparum* at delivery and presence of placental malaria infection.

**Table 1:** Haematological and parasitological data among 576 mothers and their newborns

**Parameters**	**Mothers (peripheral blood)**	**Newborns (cord blood)**
Haemoglobin (g/dl)	11.3 (± 1.5)	14.5 (± 2.0)
mean (± SD) ^a^		
Anaemia	224 (39.1)	351 (61.5)
n (%) ^b^		
*P. falciparum* prevalence	49 (8.5)	5 (0.9)
n (%) ^c^		
Parasite density ^d^	556 (159–9990)	367 (235–1492)

^a^ 3 (5) missing values for mothers (newborns).

^b^ anaemia defined as Hb < 11 g/dl (mothers) and Hb < 15 g/dl (newborns).

^c^ 2 missing values.

^d^ median parasite density (/μl) (25^th^-75^th^ percentiles), zero values excluded.

#### Maternal *IL-4*, *IL-10* and *IL-13* gene polymorphisms

**Figure 1:**
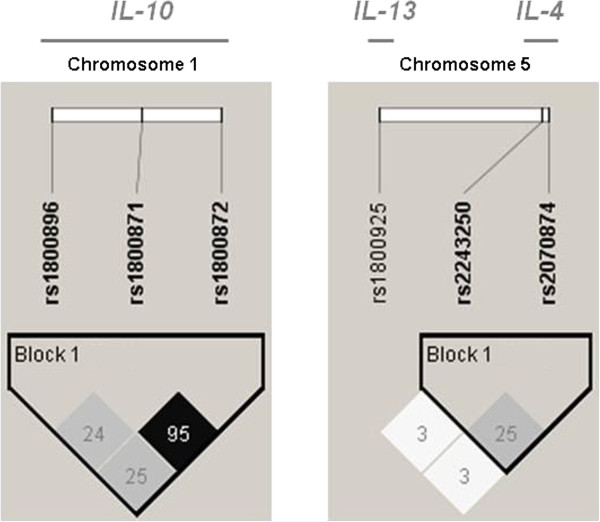
**Patterns of linkage disequilibrium between *IL-10 *SNPs and between *IL-4 *and *IL-13 *SNPs.** High pairwise LD (*r*^2^) between markers is illustrated with dark shading. The *r*^2^ values (× 100) for the marker pairs are listed in the corresponding boxes.

Cytokine gene polymorphisms were determined for the whole group of 576 mothers. No statistically significant deviation from Hardy-Weinberg equilibrium was observed for any polymorphism (all *P*-values > 0.44, Table 2). The heterozygous carriage was predominant (at least 50%), except for *IL-4*_*−590*_ and *IL-10*_*−1082*_. Since the *IL-13*_*−1055*_ SNP presented virtually no LD with the two *IL-4* SNPs in position −590 and +33 along the chromosome 5 (r^2^ = 0.03 with both SNPs, Figure 1), only haplotypes including the two *IL-4* SNPs were reconstructed and the *IL-13* SNP was treated independently in statistical analyses. By contrast, the three *IL-10* SNPs located on chromosome 1 belong to a single block of LD (Figure 1) and were thus combined into haplotypes. On the basis of literature data, the particular *IL-4*_−590_*T/IL-4_+33_*T and *IL-10*_−1082_*A/IL-10_−819_*T/IL-10_−592_*A haplotypes were selected, in order to examine their relationships with biological and immunological parameters. This allowed to consider three groups of mothers bearing either two copies (n = 140), one copy (n = 291) or no copy (n = 145) of *IL-4*_−590_*T/IL-4_+33_*T haplotype (referred to as *IL4-TT* haplotype). Similarly, three groups of mothers were defined, who carried either two copies (n = 87), one copy (n = 293) or no copy (n = 196) of *IL-10*_−1082_*A/IL-10_−819_*T/IL-10_−592_*A haplotype (referred to as *IL10-ATA* haplotype).

**Table 2:** Distribution of cytokine gene polymorphisms in the study population of 576 mothers

**Gene**	**SNPs**	**Location in the gene**	**Genotype**	**Frequency in the study population**	**Hardy-Weinberg equilibrium test χ**^**2**^**(*P*-value, df = 2)**
*IL-4*	rs2243250	*−590 C/T*	*CC*	25 (4%)	χ ^2^ = 0.03 (*P* = 0.98)
			*CT*	193 (34%)	
			*TT*	358 (62%)	
	rs2070874	*+33 C/T*	*CC*	145 (25%)	χ ^2^ = 0.08 (*P* = 0.96)
			*CT*	290 (50%)	
			*TT*	141 (25%)	
*IL-10*	rs1800896	*−1082 G/A*	*GG*	47 (8%)	χ ^2^ = 0.63 (*P* = 0.73)
			*GA*	223 (39%)	
			*AA*	306 (53%)	
	rs1800871 ^a^	*−819 C/T*	*CC*	184 (33%)	χ ^2^ = 1.09 (*P* = 0.58)
			*CT*	281 (51%)	
			*TT*	90 (16%)	
	rs1800872	*−592 C/A*	*CC*	191 (33%)	χ ^2^ = 1.64 (*P* = 0.44)
			*CA*	294 (51%)	
			*AA*	91 (16%)	
*IL-13*	rs1800925	*−1055 C/T*	*CC*	186 (32%)	χ ^2^ = 1.48 (*P* = 0.48)
			*CT*	295 (51%)	
			*TT*	95 (17%)	

^a^ 21 missing values.

**Figure 2:**
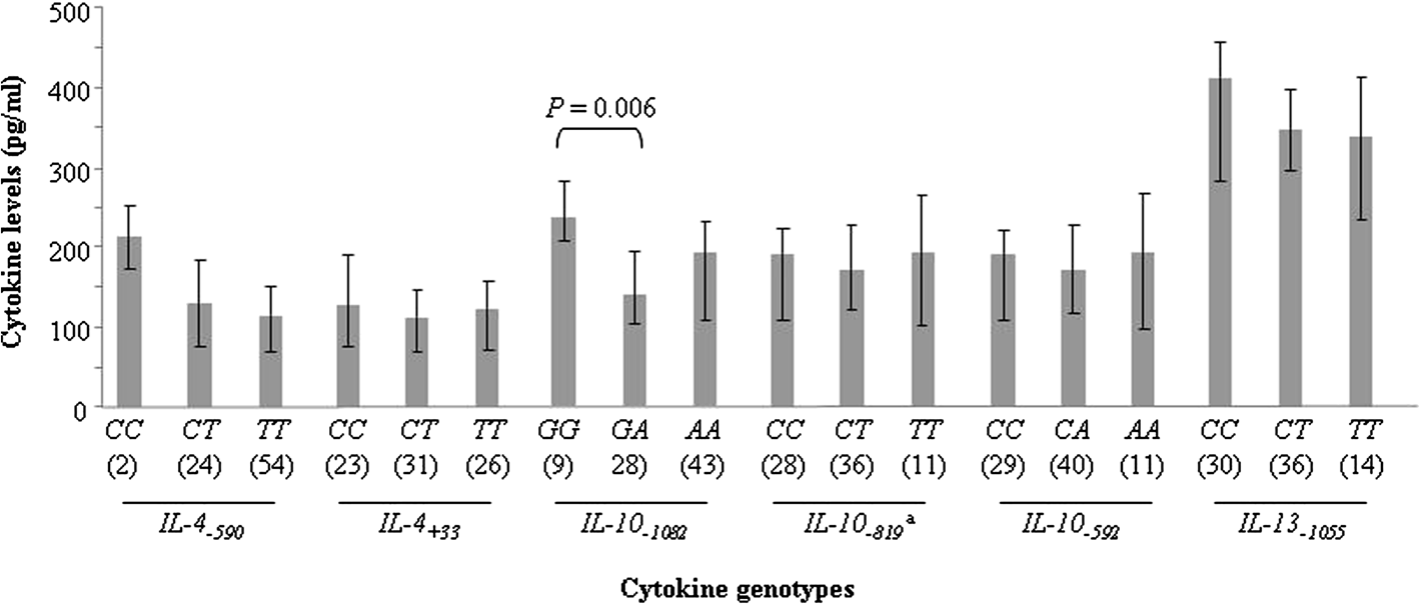
**Plasma levels of cytokines according to cytokine genotypes, in 80 mothers at delivery.**^a^ 5 missing values. Boxes delimit median values and bars denote the 25^th^ and 75^th^ percentiles. Differences in cytokine levels between genotypes were examined for each cytokine variant with the Mann–Whitney *U* test.

**Figure 3:**
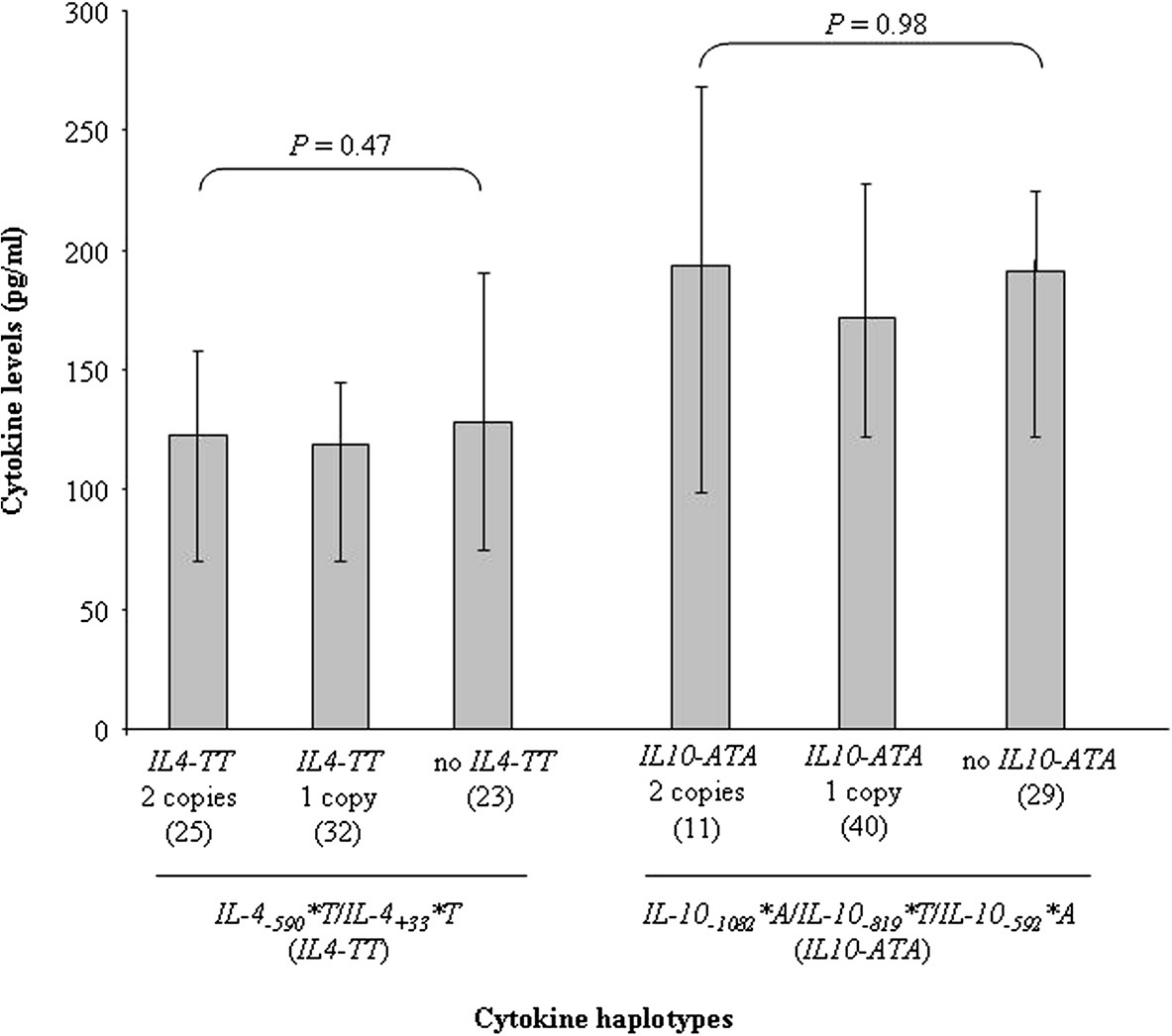
**Plasma levels of cytokines according to cytokine haplotypes, in 80 mothers at delivery.** Boxes delimit median values and bars denote the 25^th^ and 75^th^ percentiles. Differences in cytokine levels between haplotypes were examined with the Kruskal-Wallis test.

Genotype-dependent plasma levels of cytokines are presented in Figure 2 in a sub-sample of 80 mothers, where it appears that similar IL-4, IL-10 or IL-13 levels were secreted in the plasma of mothers harboring each of the three genotypes of the corresponding cytokine gene polymorphisms, except for *IL-10*_*−1082*_, for which mothers with the *GA* genotype presented lower IL-10 plasma levels than their counterparts with the *GG* genotype (*P* = 0.006). But this difference did not impact the cytokine levels among the different *IL-4* and *IL-10* haplotype groups, as illustrated in Figure 3.

#### Association between maternal cytokine gene polymorphisms and biological data of mothers

Maternal haemoglobin levels at delivery were impacted by *IL-4* gene polymorphisms (Table 3, on the basis of the results of the univariate analysis presented in the Additional file 1). Indeed, presence of the *IL-4*_+33_*T allele in genotypes *CT* and *TT* was associated with higher haemoglobin levels than those found in mothers carrying the *CC* genotype (*P* = 0.03). This observation was reinforced when examining the association of the *IL4-TT* haplotype under a dominant effect (*P* = 0.03). The same associations remain valid when considering maternal anaemia, defined by an Hb level < 11 g/dl, where it appeared that *IL-4*_*+33*_*CT* and *TT* carriers as well as carriers of one or two copies of *IL4-TT* haplotype were less at risk of anaemia than *IL-4*_*+33*_*CC* and non *IL4-TT* carriers, respectively (both analyses, OR = 0.66, *P* = 0.04).

Village localization, young mother’s age, primigravidity and *IL-4* gene polymorphisms were associated with placental malaria infection under a dominant model (Table 3). Indeed, mothers harboring either the *IL-4*_−590_*T allele in genotypes *CT* and *TT* or the *IL-4*_+33_*T allele in genotypes *CT* and *TT* were less numerous to present placental malaria infection at delivery than mothers with *CC* genotype (OR = 0.36, *P* = 0.03 and OR = 0.54, *P* = 0.03, respectively). The same pattern was observed for the *IL4-TT* haplotype: a lesser prevalence of placental malaria infection was confirmed in mothers carrying one or two copies of the *IL4-TT* haplotype when compared to non *IL4-TT* haplotype carriers (OR = 0.54, *P* = 0.03).

Village localization, primigravidity and *IL-10*_*−1082*_ polymorphism were associated with placental malaria infection under a recessive model, with more *IL-10*_*−1082*_*AA* mothers presenting placental malaria infection at delivery in comparison to *IL-10*_*−1082*_*GG* and *GA* mothers (OR = 1.95 *P* = 0.02) (Table 3). Due to the strong correlation between circulating parasite densities and prevalence of placental infection at delivery, maternal age, village, primigravidity and the same *IL-10*_*−1082*_*AA* genotype were positively associated under a recessive model with the level of circulating parasite densities (OR [CI_95_] = 2.34 [1.23-4.48], *P* = 0.01).

**Table 3:** Association between maternal cytokine gene polymorphisms and mothers’ biological parameters: multivariate analysis

**Maternal cytokine gene (n)**	**Haemoglobin (g/dl) (SD)**^**a**^	***P***^**b**^	**Anaemia OR [CI**_**95**_**]**^**c**^	***P***^**b**^	***P. falciparum*placenta infection OR [CI**_**95**_**]**^**d**^	***P***^**e**^
*IL-4*_*−590*_ genotypes:						
*CC* (25)	11.2 (0.3)		1			
*CT* (193)	11.1 (0.3)	0.88	1.13 [0.48-2.65]	0.77		
*TT* (358)	11.3 (0.3)	0.65	0.87 [0.38-1.98]	0.73	OR _*CT* and *TT* vs. *CC*_ = 0.36 [0.14-0.93]	**0.03**
*IL-4*_*+33*_ genotypes:						
*CC* (145)	11.3 (0.2)					
*CT* and *TT* (431)	11.6 (0.1)	**0.03**	OR _*CT* and *TT* vs. *CC*_ = 0.66 [0.44-0.97]	**0.04**	OR _*CT* and *TT* vs. *CC*_ = 0.54 [0.31-0.94]	**0.03**
*IL-4*_*−590*_*/IL-4*_*+33*_ haplotypes:						
No *IL4-TT* (145)	11.3 (0.2)					
1 or 2 copies *IL4-TT* (431)	11.6 (0.1)	**0.03**	OR _1 or 2 copies *IL4-TT* vs. no *IL4-TT*_ = 0.66 [0.44-0.97]	**0.04**	OR _1 or 2 copies *IL4-TT* vs. no *IL4-TT*_ = 0.54 [0.31-0.94]	**0.03**
*IL-10*_*−1082*_ genotypes:						
*GG* (47)	11.4 (0.2)		1			
*GA* (223)	11.3 (0.2)	0.53	1.01 [0.52-1.94]	0.98		
*AA* (306)	11.3 (0.2)	0.59	1.23 [0.65-2.34]	0.52	OR _*AA* vs *GG* and *GA*_ = 1.95 [1.11-3.42]	**0.02**

^a^ 3 missing values.

^b^ analysis adjusted on village.

^c^ anaemia defined as Hb < 11 g/dl.

^d^ 13 missing values.

^e^ analysis adjusted on parity and village.

*P* values < 0.05 are in bold.

#### Association between maternal cytokine gene polymorphisms and biological data of newborns

Newborn’s anaemia, defined by an Hb level < 15 g/dl, but not cord haemoglobin levels considered as a continuous variable, was impacted by *IL-10* gene polymorphisms (Table 4, on the basis of the results of the univariate analysis presented in the Additional file 2). Indeed, maternal anaemia, young maternal age and *IL-10*_*−819*_ polymorphism were associated with newborn’s anaemia under a dominant model: the presence of the *IL-10*_−819_*T allele in the *CT* and *TT* genotypes was related to a higher risk of newborn’s anaemia compared to its absence in *CC* carriers (OR = 1.46, *P* = 0.04). Combined to a borderline effect towards a higher risk of anaemia in infants born to *IL-10*_*−1082*_*GA* and *AA* mothers compared to *GG* ones (OR = 1.81, *P* = 0.06), this effect persisted in infants issued from mothers carrying one or two copies of *IL10-ATA* haplotype compared to mothers without the *IL10-ATA* haplotype (OR = 1.45, *P* = 0.04).

Birthweight, either considered as a continuous variable or dichotomized into less or more than 2500 g, was not influenced by the maternal cytokine gene polymorphisms under study.

**Table 4:** Association between maternal cytokine gene polymorphisms and newborns’ biological parameters: multivariate analysis

**Maternal cytokine gene (n)**	**Haemoglobin (g/dl) (SD)**^**a**^	***P***^**b**^	**Anaemia OR [CI**_**95**_**]**^**c**^	***P***^**d**^
*IL-10*_*−1082*_ genotypes:				
*GG* (47)	15.1 (0.3)			
*GA* and *AA* (529)	14.6 (0.3)	0.07	OR _*GA* and *AA* vs *GG*_ = 1.81 [0.98-3.32]	0.06
*IL-10*_*−819*_ genotypes:				
*CC* (191)	14.8 (0.2)			
*CT* and *TT* (385)	14.6 (0.2)	0.14	OR _*CT* and *TT* vs *CC*_ = 1.46 [1.02-2.10]	**0.04**
*IL-10*_*−592*_ genotypes:				
*CC* (191)	14.7 (0.1)			
*CA* and *AA* (385)	14.4 (0.2)	0.13	OR _*CA* and *AA* vs *CC*_ = 1.36 [0.95-1.95]	0.09
*IL-10*_*−1082*_*/IL-10*_*−819*_*/IL-10*_*−592*_ haplotypes*:*				
No *IL10-ATA* (196)	14.8 (0.2)			
1 or 2 copies *IL10-ATA* (380)	14.6 (0.2)	0.13	OR _1 or 2 copies *IL10-ATA* vs. no *IL10-ATA*_ = 1.45 [1.02-2.08]	**0.04**

^a^ 5 missing values.

^b^ analysis adjusted on maternal anaemia (Hb < 11 g/dl) and low birthweight (< 2500 g).

^c^ newborn’s anaemia defined as Hb < 15 g/dl.

^d^ analysis adjusted on maternal anaemia and age.

*P* values < 0.05 are in bold.

### Association between maternal cytokine gene polymorphisms and antibody responses of mothers

**Figure 4:**
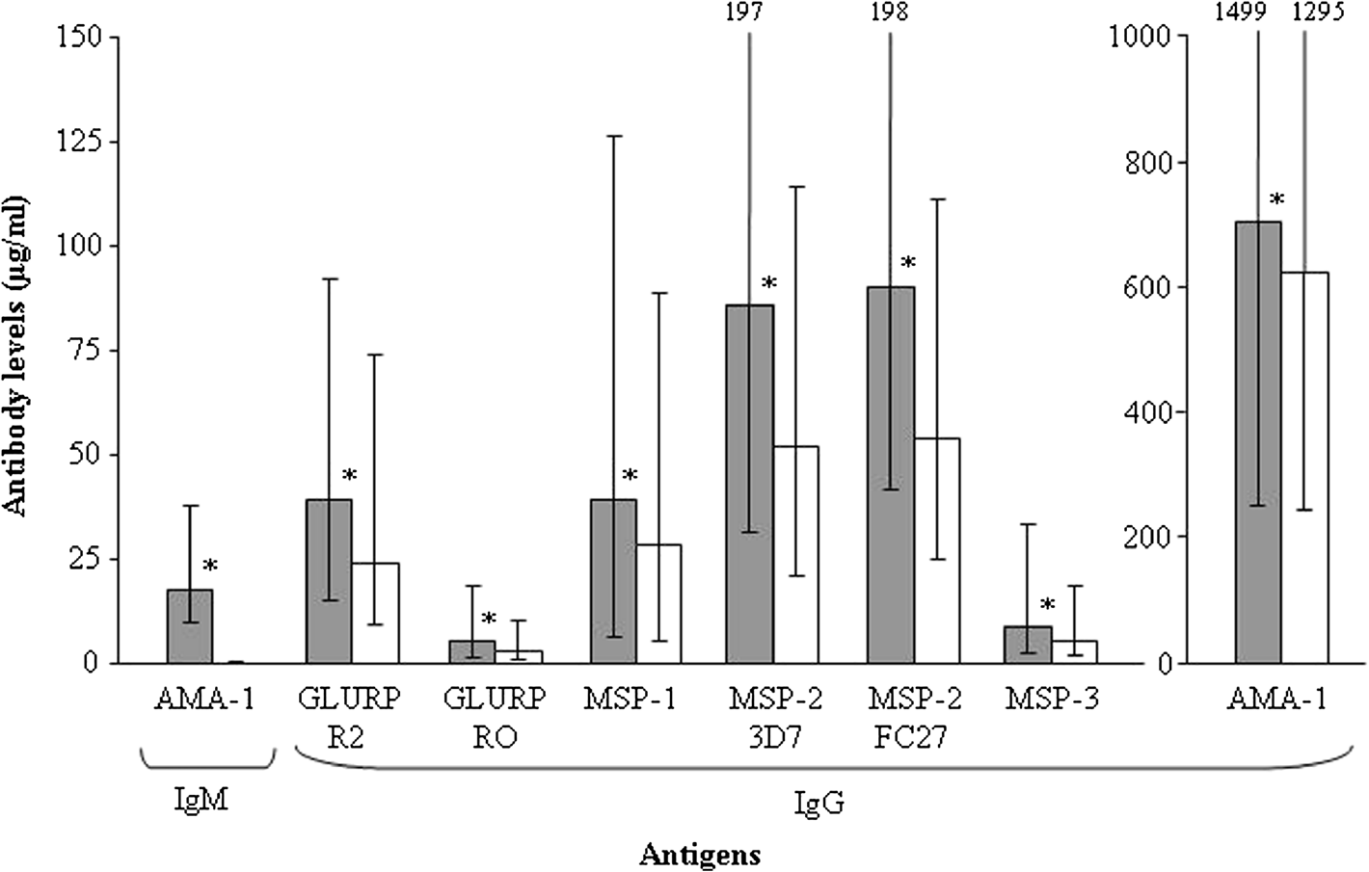
**Antibody levels to *P. falciparum *recombinant proteins in 576 mothers at delivery and their newborns.** Boxes delimit median values and bars denote the 25^th^ and 75^th^ percentiles. Asterisks (*) indicate that mothers have higher antibody levels than their newborns (Mann Whitney *U*-test, all *P* < 0.02) Symbols used for bars: mothers (grey) and infants (white).

IgG levels to the seven *P. falciparum* recombinant proteins under investigation are illustrated in Figure 4, as well as IgM to AMA-1. In all cases, specific antibody levels were higher in the circulating blood of mothers at delivery than in the cord blood of their newborns (all *P* < 0.02). The AMA-1 recombinant protein elicited the highest antibody levels in both mother and child groups.

MSP-1, MSP-2/3D7 and MSP-2/FC27 were the only antigens for which specific antibody responses of mothers were clearly associated with cytokine gene polymorphisms (Table 5 on the basis of the results of the univariate analysis presented in the Additional file 3). More precisely, *IL-4*_*−590*_*TT* carriers presented lower IgG levels to MSP-1 (*P* = 0.02) and MSP-2/3D7 (*P* = 0.03) than mothers with *CC* and *CT* genotypes. The same *IL-4*_*−590*_ polymorphism was related to the antibody response to MSP-2/FC27 under an additive model, with decreasing IgG levels from mothers bearing *CC* to *CT* and *TT* genotypes (*P* = 0.02). Finally, IgG levels to MSP-2/3D7 were higher for mothers with the *IL-10*_*−1082*_*AA* genotype than for those with *GG* and *GA* genotypes (*P* = 0.04).

**Table 5:** **Association between maternal cytokine gene polymorphisms and antibody levels to *P. falciparum *antigens in mothers: multivariate analysis**

**Antibody response**	**Maternal cytokine gene (n)**	**Antibody levels (μg/ml)**^**a**^**(SD)**	***P***
IgM to AMA-1			
	*IL-10*_*−1082*_ genotypes:		
	*GG* (47)	13.9 (3.3)	
	*GA* (223)	17.0 (3.5)	0.08 ^b^
	*AA* (306)	20.0 (3.7)	
IgG to MSP-1			
	*IL-4*_*−590*_ genotypes:		
	*CC* and *CT* (218)	49.0 (5.5)	
	*TT* (358)	32.8 (7.0)	**0.02**^c^
IgG to MSP-2/3D7			
	*IL-4*_*−590*_ genotypes:		
	*CC* and *CT* (218)	95.5 (10.3)	
	*TT* (358)	70.3 (11.9)	**0.03**^d^
	*IL-10*_*−1082*_ genotypes:		
	*GG* and *GA* (270)	68.2 (6.2)	
	*AA* (306)	92.9 (12.2)	**0.04**^d^
IgG to MSP-2/FC27	*IL-4*_*−590*_ genotypes:		
	*CC* (25)	147.7 (61.7)	
	*CT* (193)	98.0 (62.7)	
	*TT* (358)	85.4 (62.8)	**0.02**^b^
IgG to GLURP-R2 ^e^			
	*IL-4*_*−590*_ genotypes:		
	*CC* (25)	23.2 (6.9)	
	*CT* and *TT* (551)	38.3 (7.6)	0.05 ^d^
	*IL-10*_*−1082*_*/IL-10*_*−819*_*/IL-10*_*−592*_ haplotypes:		
	No or 1 copy *IL10-ATA* (489)	38.6 (3.0)	
	2 copies *IL10-ATA* (87)	24.6 (7.6)	0.07 ^d^

^a^ median value.

^b^ analysis adjusted on village.

^c^ analysis adjusted on presence of *P. falciparum* circulating parasites in mothers and presence of placental parasites.

^d^ analysis adjusted on presence of placental parasites.

^e^ 5 missing values.

*P* values < 0.05 are in bold.

### Association between maternal cytokine gene polymorphisms and antibody responses of newborns

Only cord blood IgG levels directed to AMA-1 were impacted by maternal cytokine gene polymorphisms (Table 6, on the basis of the results of the univariate analysis presented in the Additional file 4). Namely, an association involved IgG levels to AMA-1 and *IL-10*_*−1082*_ under a recessive effect, with higher IgG levels in newborns from *IL-10*_*−1082*_*AA* vs. *GG* and *GA* mothers (*P* = 0.04). In contrast, maternal *IL-13*_*−1055*_ was associated with lower IgG levels to AMA-1 under a recessive effect, concerning newborns from *IL-13*_*−1055*_*TT* vs. *CC* and *CT* mothers (*P* = 0.03).

**Table 6:** **Association between maternal cytokine gene polymorphisms and antibody levels to *P. falciparum *antigens in newborns: multivariate analysis**

**Antibody response**	**Maternal cytokine gene (n)**	**Antibody levels (μg/ml)**^**a**^**(SD)**	***P***
IgG to AMA-1			
	*IL-10*_*−1082*_ genotypes:		
	*GG* and *GA* (270)	1497.3 (214.0)	
	*AA* (306)	1638.9 (68.3)	**0.04**^b^
	*IL-13*_*−1055*_ genotypes:		
	*CC* and *CT* (481)	1596.0 (230.8)	
	*TT* (95)	1444.0 (69.9)	**0.03**^b^
IgG to MSP-2/3D7			
	*IL-10*_*−1082*_ genotypes:		
	*GG* and *GA* (270)	46.0 (4.1)	
	*AA* (306)	57.6 (6.5)	0.08 ^c^
IgG to GLURP-R0			
	*IL-10*_*−592*_ genotypes:		
	*CC* and *CA* (485)	3.2 (0.3)	
	*AA* (91)	2.2 (0.5)	0.06 ^c^
	*IL-10*_*−1082*_*/IL-10*_*−819*_*/IL-10*_*−592*_ haplotypes:		
	No or 1 copy *IL10-ATA* (489)	3.2 (0.3)	
	2 copies *IL10-ATA* (87)	2.2 (0.5)	0.05 ^c^

^a^ median value.

^b^ analysis adjusted on maternal age, parity and estimated exposure to malaria transmission during pregnancy.

^c^ analysis adjusted on presence of *P. falciparum* circulating parasites in mothers.

*P* values < 0.05 are in bold.

## Discussion

The present study aimed at exploring the impact of maternal cytokine gene polymorphisms on biological parameters and antimalarial antibody responses of mothers at delivery and their newborns. Cytokines involved in antibody production such as IL-4, IL-10 and IL-13 were investigated. A clear impact of *IL-4* gene polymorphisms on biological parameters of mothers at delivery was revealed. Indeed, the *IL4-TT* haplotype (one or two copies) was found negatively associated with *P. falciparum* placenta infection and maternal anaemia. The *IL-4*_*−590*_*TT* genotype was related to lower maternal IgG levels to MSP-1, MSP-2/3D7 and MSP-2/FC27 than the *CC* and *CT* genotypes. The maternal *IL-4* gene polymorphism did impact neither biological parameters nor antibody responses of the newborns. *IL-10*_*−1082*_*AA* genotypes were positively associated with both *P. falciparum* placenta infection and circulating parasites in mothers. Similarly, *IL-10*_*−819*_*CT* and *TT* genotypes as well as the *IL10-ATA* haplotype (one or two copies) were positively related to anaemia in newborns. Concerning antibody responses, *IL-10*_*−1082*_*AA* genotypes were associated with higher IgG levels to MSP-2/3D7 and AMA-1 in mothers and in newborns, respectively. The only association involving *IL-13*_*−1055*_ concerned the IgG response to AMA-1 in cord blood, with lower levels in newborns from mothers with the *TT* vs. *CC* and *CT* genotypes. *IL-4* and *IL-10* haplotypes were not related to plasma cytokine levels, probably due to the small sample size of the sub-population in which cytokine levels were measured.

Around 40% of the mothers and more than 60% of the newborns presented anaemia. The relationships between anaemia during pregnancy and birth outcomes were studied in depth in this cohort by Koura et al., who noticed that these prevalence rates were in agreement with those already recorded in Benin, and observed that newborn’s anaemia was related to maternal anaemia [50].

Our finding of a lower prevalence of placental malaria infection in mothers with either the *IL-4*_*−590*_ or *IL-4*_*+33*_*CT* and *TT* genotypes, or still the *IL4-TT* haplotype, is in accordance with recent results indicating reduced malaria risk in *IL4-TT* carriers from Indian tribal populations [51]. On the contrary, the *IL-4*_−590_*T allele was related to high prevalence of *P. falciparum* infection in asymptomatic Fulani of Mali, but not in a sympatric group of Dogon [19]. The lesser prevalence of placental parasites in mothers carrying one or two copies of the *IL4-TT* haplotype was consistent with the higher haemoglobin values (and decreased risk of anaemia) observed in these mothers at delivery, probably due to a lesser impact of malaria-induced anaemia. A complementary explanation would be that IL-4 is known for its role in the promotion of erythropoiesis [52]. It would be coherent that these favorable maternal condition should be accompanied by strong antimalarial antibody responses, as it has been already demonstrated for specific IgG in Fulani individuals with the *IL-4*_−590_*T allele [18]. This would be in agreement with a previous observation made in malaria-infected individuals, of an association between the activation of IL-4 producing T-cell subsets and the production of antimalarial specific antibodies [11]. Those antibodies would reflect an effective response to infecting parasites [53], allowing to combat infection and therefore to prevent its evolution toward high parasite densities and sequestration into the placenta. Nonetheless, in the present study, the *IL-4*_*−590*_*TT* genotype was uniformly associated negatively with maternal IgG levels to MSP-1, MSP-2/3D7 and MSP-2/FC27. The most obvious hypothesis for this observation rests on a decreased induction of antimalarial antibodies in *IL-4*_*−590*_*TT* mothers as a result of a lighter weight of *P. falciparum* placenta infection in these mothers. It should be reminded that the Beninese women under study cumulated previous malaria experience, and/or that co-infection with other parasitic or bacterial pathogens not investigated in the present study may have occurred, thereby preventing a clear relationship. Some regulatory factors placed under genetic control and in LD with *IL-4* gene polymorphisms may also impact the data [54]. In fact, studies on the relationships between *IL-4* gene polymorphisms and IgG levels are limited and conflicting [18,55]. The Th2 cytokine induces Ig class switching from IgM/IgG to IgE [9,10]. IL-4 has been incriminated in the aggravation of cerebral malaria due to its role in an increase in parasite mass [56], in an infiltration by monocytes, basophils, and eosinophils and an increase in parasite sequestration [57]. In the context of our study, independently of any morbidity linked to malaria, no biological or immunological advantage could be deduced for the newborns from the *IL-4* genetic characteristics of their respective mothers. Comparatively to IgG which are mainly of maternal origin in the cord blood due to the existence of a passive transfer of antibodies during the pregnancy, IgM do not cross the placenta and are therefore synthesized by the fetus, as a result of a sensitization to malaria antigens *in utero*[58,59]. Nevertheless, the low levels and prevalence rates of newborns’ IgM to AMA-1 hampered any observation in link with maternal cytokine gene polymorphisms.

As for *IL-4*, data from the literature indicate that *IL-10* gene polymorphisms need to be analyzed combined into haplotypes and no longer at the limiting scale of single nucleotide polymorphisms. We thus studied the effects of the candidate *IL-10*_*−1082*_*G/A, IL-10*_*−819*_*C/T* and *IL-10*_*−592*_*C/A* polymorphisms simultaneously, through a haplotype analysis, in relation to the biological and immunological data recorded in both the mothers and their newborns. As for *IL-4*, were only considered the final results issued from a multivariate analysis taking into account specific maternal parameters such as age, parity, residence place, the estimated entomological inoculation rate during the whole pregnancy period and/or the presence of circulating parasites as well as placental parasites at delivery. Special attention was paid to the *IL-10*_−1082_*A/IL-10_−819_*T/IL-10_−592_*A (*IL10-ATA*) haplotype carriers, assimilated to low IL-10 producers [60,61]. It emerges from this study that infants born from *IL10-ATA* (and *IL-10*_*−819*_*CT* and *TT*) mothers inherit poorer physiological conditions than others, with a greater risk of anaemia, possibly in relation to the greater risk of placenta malarial infection as well as presence of circulating parasites observed in *IL-10*_*−1082*_*AA* mothers.

IL-10 is an important immune modulating factor with anti-inflammatory activities that lead to inhibition of the activation of antigen-presenting cells, resulting in their reduced ability to induce T cell responses. But IL-10 has also some pro-inflammatory activity since it promotes activation and differentiation of B cells and induces immunoglobulin synthesis [62]. It is therefore expected that low IL-10 producers such as the *IL10-ATA* carriers present lower antibody levels compared to no *IL10-ATA* carriers. For example, a previous study showed that the *IL-10*_−1082_*A/IL-10_−592_*A haplotype was associated with decreased specific IgE and IgG4 levels [23]. But conversely to this expectation, *IL-10*_*−1082*_*AA* mothers of the present study had higher IgG to MSP-2/3D7 than *GG* and *GA* ones. This pattern of response found an immunological echo in the newborns, as those born from mothers with the *IL-10*_*−1082*_*AA* genotype presented higher IgG levels to AMA-1 compared to newborns from *GG* and *GA* mothers. Two hypotheses can be put forward to explain this observation. Firstly, the lower degree of control of *P. falciparum* infection in *IL-10*_*−1082*_*AA* mothers may result in a strongest antibody response in both mothers and their newborns (via the *in utero* transfer of maternal IgG and/or the production of intrinsic IgG in response to parasitic soluble antigens encountered *in utero*). Secondly, there is no dogma regarding a decreased IL-10 production in individuals with *IL10-ATA* haplotype, as the combination of a particular *IL-1* genotype with the *IL10-ATA* haplotype was shown to result in increased IL-10 plasma levels in healthy Finnish individuals, demonstrating the importance of the individual genetic background [63].

Turner et al. demonstrated a difference in IL-10 secretion in association with the presence or absence of of the *IL-10*_−1082_*A allele in the human *IL-10* promoter, after stimulation of peripheral blood mononuclear cells [22]. This is in accordance with our observation of lower IL-10 plasma levels in mothers with the *IL-10*_*−1082*_*GA* genotype compared to those with the *GG* genotype. Nevertheless, no difference in the plasma IL-10 levels according to *IL-10* haplotypes was recorded in the sub-sample of 80 mothers for whom cytokine levels were measured. It should be noted that individual differences in the levels of the IL-10 measured at a specific moment may not only result from host genetic factors predisposing to high or low production, but also for a great part from the physiological condition at that time, as well as from global immunity.

In Thailand, the functional *IL-13*_−1055_*T allele has been shown to enhance resistance to severe malaria through the alteration of IL-13 production [16]. The same allele has been associated with resistance to *S. mansoni*[64,65] and inversely with susceptibility to *S. haematobium* infection [66]. Isnard et al. established that the *IL-13*_*−1055*_*C/T* polymorphism is in LD with other polymorphisms [66], suggesting that only weak effects may be revealed when investigating the single polymorphism in relation to clinical or immunological phenotypes. This may explain why *IL-13*_*−1055*_*C/T* alleles were not found associated with any parameter in the present study, except lower IgG levels to AMA-1 in newborns from *IL-13*_*−1055*_*TT* vs. *CC* and *TT* mothers. It would be unwise to comment this observation, as it did not present any biological corollary (either in mothers or newborns) in the present study.

Besides the main results, it is interesting to note that the distribution of the cytokine genotypes in our study population from south Benin (most participants belonging to the Tori ethnic group) is similar to that observed in the Yoruba (YRI) sample from the 1000 Genomes project [67], located in the neighboring country (Nigeria), and the opposite of the one observed in the European (CEU) sample of the same database (data not shown). This distribution profile may suggest a selective genetic advantage conferred by the secular pressure exerted by infectious diseases such as malaria [68].

## Conclusion

This study shows that *IL-4* and *IL-10* gene polymorphisms are likely to play a role in the regulation of mothers’ and newborns’ control of malaria infection. This is the first demonstration of an impact of maternal *IL-10* gene polymorphisms on the newborn specific IgG production, opening hypotheses about the consequences that this may have later in the life of the child for the elaboration of his own IgG responses. These results may provide a contribution to a better understanding of the immunopathogenic mechanisms underlying maternal-fetal interactions. Further studies are required to strengthen the knowledge of the impact of maternal gene polymorphisms on the immunoglobulin isotypes synthesized by the fetus and thereafter the infant.

## Abbreviations

AMA: Apical membrane antigen; ELISA: Enzyme-linked immunosorbent assay; GLURP: Glutamate rich protein; Ig: Immunoglobulin; IL: Interleukin; LD: Linkage disequilibrium; MSP: Merozoite surface protein; OR: Odds ratio; PCR: Polymerase chain reaction; SNP: Single nucleotide polymorphism.

## Competing interests

The authors declare no conflict of interest.

## Authors’ contributions

LAG, GA and MNF designed the study. LAG, GA, MNF, DC, CD, LPA, AC, FB and MA recruited the children and the adults and conducted the field work. LR, NJ, BD and AC performed microscopic reading while LAG, DC, BA, CD, LR and MNF realized the laboratory assays. LAG, GA, SA and MNF analyzed the data and drafted the manuscript. The final manuscript was read and approved by all authors.

## Pre-publication history

The pre-publication history for this paper can be accessed here:

http://www.biomedcentral.com/1471-2334/13/215/prepub

## Supplementary Material

Additional file 1 **Association between maternal cytokine gene polymorphisms and mothers’ biological parameters (n = 576): univariate analysis.** This table summarizes the results of the univariate analysis performed for examining differences between maternal cytokine genotypes or haplotypes and biological parameters (haemoglobin, prevalence of circulating *P. falciparum* parasites, prevalence of *P. falciparum* placenta infection) of mothers. Click here for file

Additional file 2 **Association between maternal cytokine gene polymorphisms and newborns’ biological parameters (n = 576): univariate analysis.** This table summarizes the results of the univariate analysis performed for examining differences between maternal cytokine genotypes or haplotypes and biological parameters (birthweight, haemoglobin) of newborns. Click here for file

Additional file 3 **Association between maternal cytokine gene polymorphisms and mothers’ antibody levels to *P. falciparum *antigens (n = 576): univariate analysis.** This table summarizes the results of the univariate analysis performed for examining differences between maternal cytokine genotypes or haplotypes and mothers’ IgG levels to 7 recombinant proteins of *P. falciparum* asexual stage antigens as well as IgM levels to 1 of these antigens. Click here for file

Additional file 4 **Association between maternal cytokine gene polymorphisms and newborns’ antibody levels to *P. falciparum *antigens (n = 576): univariate analysis.** This table summarizes the results of the univariate analysis performed for examining differences between maternal cytokine genotypes or haplotypes and newborns’ IgG levels to 7 recombinant proteins of *P. falciparum* asexual stage antigens as well as IgM levels to 1 of these antigens. Click here for file
