# Supplementary material for: Association of IL-4 and IL-10 maternal haplotypes with immune responses to P. falciparum in mothers and newborns
Source: BMC Infect Dis. 2013 May 13;13:215. doi: 10.1186/1471-2334-13-215 (PMC3679728; doi:10.1186/1471-2334-13-215)
Supplement: Additional file 1 — Association between maternal cytokine gene polymorphisms and mothers’ biological parameters (n = 576): univariate analysis. This table summarizes the results of the univariate analysis performed for examining differences between maternal cytokine genotypes or haplotypes and biological parameters (haemoglobin, prevalence of circulating P. falciparum parasites, prevalence of P. falciparum placenta infection) of mothers. [file 1471-2334-13-215-S1.doc]

**Additional file 1:** Association between maternal cytokine gene polymorphisms and mothers’ biological parameters (n = 576): univariate analysis

| *Maternal cytokine gene (n)* | |  | *Mothers’ biological parameters* | | | | | |
| --- | --- | --- | --- | --- | --- | --- | --- | --- |
|  |  |  | *Hemoglobin*  *(g/dl) ^a, b^* | *P ^c^* | *P. falciparum prevalence*  *n (%) ^d^* | *P ^e^* | *P. falciparum placenta infection*  *n (%) ^f^* | *P ^e^* |
| *IL-4_-590_* genotypes: | |  |  |  |  |  |  |  |
|  | *CC* (25) |  | 11.1 (10.6-11.7) |  | 3 (0.5) |  | 7 (1.2) |  |
|  | *CT* (193) |  | 11.1 (10.3-12.1) | 0.26 | 18 (3.1) | 0.69 | 18 (3.2) | **0.02** |
|  | *TT* (358) |  | 11.4 (10.5-12.2) |  | 28 (4.9) |  | 38 (6.7) |  |
| *IL-4_+33_* genotypes: | |  |  |  |  |  |  |  |
|  | *CC* (145) |  | 11.0 (10.1-11.9) |  | 15 (2.6) |  | 23 (4.1) |  |
|  | *CT* (290) |  | 11.4 (10.4-12.2) | **0.03** | 25 (4.3) | 0.49 | 28 (5.0) | **0.10** |
|  | *TT* (141) |  | 11.5 (10.6-12.5) |  | 9 (1.6) |  | 12 (2.1) |  |
| *IL-4_-590_/IL-4_+33_* haplotypes: | |  |  |  |  |  |  |  |
|  | *No IL4-TT* (145) |  | 11.0 (10.1-11.9) |  | 15 (2.6) |  | 23 (4.1) |  |
|  | *1 copy IL4-TT* (291) |  | 11.3 (10.4-12.2) | **0.02** | 25 (4.4) | 0.50 | 28 (5.0) | **0.10** |
|  | *2 copies IL4-TT* (140) |  | 11.6 (10.6-12.5) |  | 9 (1.6) |  | 12 (2.1) |  |
| *IL-10_-1082_* genotypes: | |  |  |  |  |  |  |  |
|  | *GG* (47) |  | 11.8 (10.5-12.3) |  | 3 (0.5) |  | 4 (0.7) |  |
|  | *GA* (223) |  | 11.3 (10.4-12.1) | 0.59 | 11 (1.9) | **0.03** | 16 (2.8) | **0.05** |
|  | *AA* (306) |  | 11.2 (10.4-12.1) |  | 35 (6.1) |  | 43 (7.6) |  |
| *IL-10_-819_* genotypes: | |  |  |  |  |  |  |  |
|  | *CC* (191) |  | 11.4 (10.4-12.3) |  | 14 (2.4) |  | 20 (3.5) |  |
|  | *CT* (293) |  | 11.2 (10.4-12.1) | 0.70 | 25 (4.3) | 0.60 | 32 (5.7) | 0.94 |
|  | *TT* (92) |  | 11.3 (10.4-12.0) |  | 10 (1.7) |  | 11 (1.9) |  |
| *IL-10_-592_* genotypes: | |  |  |  |  |  |  |  |
|  | *CC* (191) |  | 11.3 (10.4-12.3) |  | 13 (2.3) |  | 19 (3.4) |  |
|  | *CA* (294) |  | 11.2 (10.4-12.1) | 0.73 | 25 (4.3) | 0.32 | 32 (5.7) | 0.72 |
|  | *AA* (91) |  | 11.4 (10.4-12.1) |  | 11 (1.9) |  | 12 (2.1) |  |
| *IL-10_-1082_/IL-10_-819_/IL-10_-592_* haplotypes: | |  |  |  |  |  |  |  |
|  | *No IL10-ATA* (196) |  | 11.3 (10.5-12.3) |  | 14 (2.4) |  | 20 (3.6) |  |
|  | *1 copy IL10-ATA* (293) |  | 11.2 (10.4-12.1) | 0.68 | 25 (4.4) | 0.47 | 32 (5.7) | 0.85 |
|  | *2 copies IL10-ATA* (87) |  | 11.4 (10.4-12.2) |  | 10 (1.7) |  | 11 (2.0) |  |
| IL-13_-1055_ genotypes: | |  |  |  |  |  |  |  |
|  | *CC* (186) |  | 11.4 (10.4-12.4) |  | 17 (3.0) |  | 22 (3.9) |  |
|  | *CT* (295) |  | 11.3 (10.4-12.1) | 0.44 | 23 (4.0) | 0.81 | 31 (5.5) | 0.90 |
|  | *TT* (95) |  | 11.3 (10.4-12.2) |  | 9 (1.6) |  | 10 (1.8) |  |

^a^ median value (25^th^-75^th^ percentiles).

^b^ 3 missing values.

^c^ differences in biological parameters were examined with the Kruskal-Wallis test.

^d^ 2 missing values.

^e^differences in prevalence rates were examined with the Chi square test.

^f^ 13 missing values.

*P* values in bold (*P* < 0.20) correspond to variables considered in the multivariate analysis.
