# Supplementary material for: Association of IL-4 and IL-10 maternal haplotypes with immune responses to P. falciparum in mothers and newborns
Source: BMC Infect Dis. 2013 May 13;13:215. doi: 10.1186/1471-2334-13-215 (PMC3679728; doi:10.1186/1471-2334-13-215)
Supplement: Additional file 2 — Association between maternal cytokine gene polymorphisms and newborns’ biological parameters (n = 576): univariate analysis. This table summarizes the results of the univariate analysis performed for examining differences between maternal cytokine genotypes or haplotypes and biological parameters (birthweight, haemoglobin) of newborns. [file 1471-2334-13-215-S2.doc]

**Additional file 2:** Association between maternal cytokine gene polymorphisms and newborns’ biological parameters (n = 576): univariate analysis

| *Maternal cytokine gene (n)* | |  | *Newborns’ biological parameters at birth* | | | |
| --- | --- | --- | --- | --- | --- | --- |
|  |  |  | *Birth weight*  *(g) ^a, b^* | *P ^c^* | *Hemoglobin*  *(g/dl) ^a, d^* | *P ^c^* |
| *IL-4_-590_* genotypes: | |  |  |  |  |  |
|  | *CC* (25) |  | 2961 (2775-3084) |  | 13.8 (13.0-16.1) |  |
|  | *CT* (193) |  | 3005 (2708-3205) | 0.82 | 14.5 (13.5-15.6) | 0.82 |
|  | *TT* (358) |  | 2945 (2705-3270) |  | 14.5 (13.5-15.7) |  |
| *IL-4_+33_* genotypes: | |  |  |  |  |  |
|  | *CC* (145) |  | 3007 (2725-3240) |  | 14.4 (13.5-15.6) |  |
|  | *CT* (290) |  | 2945 (2705-3210) | 0.74 | 14.6 (13.5-15.6) | 0.93 |
|  | *TT* (141) |  | 2995 (2705-3265) |  | 14.5 (13.3-15.8) |  |
| *IL-4_-590_/IL-4_+33_* haplotypes: | |  |  |  |  |  |
|  | *No IL4-TT* (145) |  | 3007 (2725-3240) |  | 14.4 (13.5-15.6) |  |
|  | *1 copy IL4-TT* (291) |  | 2945 (2706-3209) | 0.75 | 14.5 (13.5-15.6) | 0.93 |
|  | *2 copies IL4-TT* (140) |  | 2993 (2705-3266) |  | 14.5 (13.3-15.8) |  |
| *IL-10_-1082_* genotypes: | |  |  |  |  |  |
|  | *GG* (47) |  | 3055 (2780-3218) |  | 15.4 (13.8-16.1) |  |
|  | *GA* (223) |  | 2966 (2748-3215) | 0.30 | 14.4 (13.5-15.6) | **0.11** |
|  | *AA* (306) |  | 2951 (2689-3223) |  | 14.5 (13.5-15.6) |  |
| *IL-10_-819_* genotypes: | |  |  |  |  |  |
|  | *CC* (191) |  | 3008 (2704-3205) |  | 14.7 (13.7-15.9) |  |
|  | *CT* (293) |  | 2935 (2705-3218) | 0.64 | 14.4 (13.4-15.3) | **0.10** |
|  | *TT* (92) |  | 2963 (2724-3258) |  | 14.3 (13.1-15.6) |  |
| *IL-10_-592_* genotypes: | |  |  |  |  |  |
|  | *CC* (191) |  | 3008 (2751-3205) |  | 14.7 (13.7-15.9) |  |
|  | *CA* (294) |  | 2930 (2705-3217) | 0.50 | 14.4 (13.4-15.4) | **0.14** |
|  | *AA* (91) |  | 2970 (2724-3278) |  | 14.3 (12.9-15.6) |  |
| *IL-10_-1082_/IL-10_-819_/IL-10_-592_* haplotypes: | |  |  |  |  |  |
|  | *No IL10-ATA* (196) |  | 3005 (2715-3205) |  | 14.4 (13.4-15.4) |  |
|  | *1 copy IL10-ATA* (293) |  | 2938 (2705-3225) | 0.66 | 14.4 (13.4-15.4) | **0.09** |
|  | *2 copies IL10-ATA* (87) |  | 2955 (2724-3278) |  | 14.7 (13.7-15.9) |  |
| IL-13_-1055_ genotypes: | |  |  |  |  |  |
|  | *CC* (186) |  | 2976 (2706-3269) |  | 14.5 (13.6-15.7) |  |
|  | *CT* (295) |  | 2976 (2721-3233) | 0.47 | 14.5 (13.3-15.6) | 0.68 |
|  | *TT* (95) |  | 2958 (2650-3186) |  | 14.5 (13.7-15.7) |  |

^a^ median value (25^th^-75^th^ percentiles).

^b^ 1 missing value.

^c^ differences in biological parameters were examined with the Kruskal-Wallis test (for genotypes).

^d^ 5 missing values.

*P* values in bold (*P* < 0.20) correspond to variables considered in the multivariate analysis.
