# Supplementary material for: Association of IL-4 and IL-10 maternal haplotypes with immune responses to P. falciparum in mothers and newborns
Source: BMC Infect Dis. 2013 May 13;13:215. doi: 10.1186/1471-2334-13-215 (PMC3679728; doi:10.1186/1471-2334-13-215)
Supplement: Additional file 3 — Association between maternal cytokine gene polymorphisms and mothers’ antibody levels to P. falciparum antigens (n = 576): univariate analysis. This table summarizes the results of the univariate analysis performed for examining differences between maternal cytokine genotypes or haplotypes and mothers’ IgG levels to 7 recombinant proteins of P. falciparum asexual stage antigens as well as IgM levels to 1 of these antigens. [file 1471-2334-13-215-S3.doc]

**Additional file 3:** Association between maternal cytokine gene polymorphisms and mothers’ antibody levels to *P. falciparum* antigens (n = 576): univariate analysis

| *Maternal cytokine gene (n)* | | *IgG (µg/ml) ^a^* | | | | | | | | | | | | | | *IgM (µg/ml) ^a^* | |
| --- | --- | --- | --- | --- | --- | --- | --- | --- | --- | --- | --- | --- | --- | --- | --- | --- | --- |
|  |  | *AMA-1* | *P ^b^* | *MSP-1* | *P* | *MSP-2/3D7* | *P* | *MSP-2/FC27* | *P* | *MSP-3* | *P* | *GLURP-R0* | *P* | *GLURP-R2 ^c^* | *P* | *AMA-1* | *P* |
| *IL-4_-590_* genotypes: | |  |  |  |  |  |  |  |  |  |  |  |  |  |  |  |  |
|  | *CC* (25) | 761 (258-1534) |  | 39 (4-51) |  | 62 (37-205) |  | 148 (57-321) |  | 18 (4-43) |  | 4 (3-16) |  | 24 (14-53) |  | 12 (9-39) |  |
|  | *CT* (193) | 747 (339-1518) | 0.37 | 52 (7-135) | **0.16** | 104 (38-211) | **0.18** | 98 (51-209) | **0.06** | 9 (2-29) | 0.57 | 7 (2-24) | 0.51 | 46 (15-114) | 0.23 | 18 (10-40) | 0.58 |
|  | *TT* (358) | 680 (222-1474) |  | 31 (7-120) |  | 80 (29-185) |  | 85 (35-185) |  | 9 (2-36) |  | 5 (2-17) |  | 38 (16-89) |  | 18 (9-36) |  |
| *IL-4_+33_* genotypes: | |  |  |  |  |  |  |  |  |  |  |  |  |  |  |  |  |
|  | *CC* (145) | 720 (255-1522) |  | 40 (6-101) |  | 108 (34-229) |  | 93 (42-200) |  | 11 (3-30) |  | 5 (2-22) |  | 47 (18-110) |  | 17 (11-38) |  |
|  | *CT* (290) | 725 (304-1546) | **0.15** | 45 (7-141) | 0.31 | 83 (30-183) | 0.45 | 91 (44-190) | 0.92 | 9 (2-37) | 0.45 | 7 (2-20) | 0.80 | 38 (15-89) | 0.22 | 17 (9-37) | 0.62 |
|  | *TT* (141) | 608 (169-1284) |  | 28 (5-112) |  | 79 (30-189) |  | 85 (37-200) |  | 8 (2-28) |  | 5 (2-17) |  | 35 (14-89) |  | 21 (10-41) |  |
| *IL-4_-590_/IL-4_+33_* haplotypes: | |  |  |  |  |  |  |  |  |  |  |  |  |  |  |  |  |
|  | *No IL4-TT* (145) | 720 (255-1522) |  | 40 (6-101) |  | 108 (34-229) |  | 93 (42-200) |  | 11 (3-30) |  | 5 (2-22) |  | 47 (18-110) |  | 17 (11-38) |  |
|  | *1 copy IL4-TT* (291) | 724 (293-1535) | 0.20 | 45 (7-140) | 0.32 | 82 (30-182) | 0.45 | 91 (43-190) | 0.93 | 10 (2-37) | 0.41 | 7 (2-20) | 0.75 | 38 (15-89) | 0.23 | 17 (9-37) | 0.57 |
|  | *2 copies IL4-TT* (140) | 628 (180-1290) |  | 28 (5-113) |  | 78 (30-190) |  | 85 (37-204) |  | 8 (2-28) |  | 5 (2-17) |  | 35 (14-89) |  | 21 (10-42) |  |
| *IL-10_-1082_* genotypes: | |  |  |  |  |  |  |  |  |  |  |  |  |  |  |  |  |
|  | *GG* (47) | 608 (254-1436) |  | 29 (6-141) |  | 121 (42-223) |  | 92 (55-216) |  | 12 (5-42) |  | 4 (2-12) |  | 38 (14-79) |  | 14 (6-33) |  |
|  | *GA* (223) | 643 (224-1260) | **0.10** | 36 (6-102) | 0.41 | 75 (28-162) | **0.06** | 85 (35-177) | **0.18** | 8 (2-28) | 0.28 | 6 (2-19) | 0.79 | 41 (20-90) | 0.84 | 17 (10-32) | **0.15** |
|  | *AA* (306) | 763 (312-1629) |  | 40 (6-151) |  | 102 (32-220) |  | 96 (46-216) |  | 9 (2-37) |  | 6 (2-20) |  | 37 (14-93) |  | 20 (10-44) |  |
| *IL-10_-819_* genotypes: | |  |  |  |  |  |  |  |  |  |  |  |  |  |  |  |  |
|  | *CC* (191) | 679 (244-1308) |  | 36 (6-124) |  | 91 (34-224) |  | 91 (41-211) |  | 8 (2-28) |  | 5 (2-17) |  | 38 (16-90) |  | 18 (10-33) |  |
|  | *CT* (293) | 714 (221-1534) | 0.33 | 40 (7-119) | 0.92 | 83 (33-178) | 0.66 | 87 (35-187) | 0.25 | 10 (2-34) | 0.67 | 7 (2-24) | **0.16** | 44 (16-96) | 0.80 | 18 (10-40) | 0.78 |
|  | *TT* (92) | 720 (401-1590) |  | 39 (7-168) |  | 78 (27-203) |  | 93 (51-219) |  | 9 (3-38) |  | 4 (2-14) |  | 29 (14-84) |  | 19 (9-44) |  |
| *IL-10_-592_* genotypes: | |  |  |  |  |  |  |  |  |  |  |  |  |  |  |  |  |
|  | *CC* (191) | 679 (244-1308) |  | 36 (6-128) |  | 90 (34-224) |  | 91 (43-214) |  | 8 (2-29) |  | 5 (2-16) |  | 38 (16-90) |  | 17 (9-33) |  |
|  | *CA* (294) | 716 (222-1548) | 0.40 | 40 (7-119) | 0.99 | 82 (30-177) | 0.67 | 86 (33-187) | **0.07** | 10 (2-36) | 0.89 | 7 (2-24) | **0.16** | 44 (15-96) | 0.88 | 18 (10-39) | 0.52 |
|  | *AA* (91) | 719 (395-1550) |  | 39 (6-172) |  | 82 (28-206) |  | 101 (51-227) |  | 8 (3-34) |  | 4 (2-14) |  | 29 (15-85) |  | 21 (10-47) |  |
| *IL-10_-1082_/IL-10_-819_/IL-10_-592_* haplotypes: | |  |  |  |  |  |  |  |  |  |  |  |  |  |  |  |  |
|  | *No IL10-ATA* (196) | 680 (242-1307) |  | 36 (6-128) |  | 90 (32-228) |  | 91 (43-210) |  | 8 (2-28) |  | 5 (2-17) |  | 38 (16-90) |  | 17 (10-33) |  |
|  | *1 copy IL10-ATA* (293) | 714 (228-1534) | 0.34 | 40 (7-119) | 0.96 | 83 (31-175) | 0.67 | 86 (33-187) | **0.08** | 10 (2-37) | 0.68 | 7 (2-23) | 0.20 | 45 (16-98) | 0.69 | 18 (10-40) | 0.79 |
|  | *2 copies IL10-ATA* (87) | 719 (395-1600) |  | 39 (7-172) |  | 80 (28-206) |  | 101 (51-244) |  | 8 (3-34) |  | 4 (2-14) |  | 27 (14-81) |  | 20 (10-42) |  |
| IL-13_-1055_ genotypes: | |  |  |  |  |  |  |  |  |  |  |  |  |  |  |  |  |
|  | *CC* (186) | 673 (260-1471) |  | 30 (5-149) |  | 98 (32-222) |  | 98 (48-228) |  | 9 (2-36) |  | 6 (2-28) |  | 43 (17-102) |  | 17 (10-36) |  |
|  | *CT* (295) | 719 (257-1528) | 0.32 | 40 (7-119) | 0.80 | 84 (32-197) | 0.28 | 91 (37-213) | 0.27 | 8 (2-33) | 0.87 | 4 (2-15) | **0.06** | 37 (14-81) | 0.46 | 17 (9-39) | 0.60 |
|  | *TT* (95) | 564 (245-1267) |  | 42 (6-91) |  | 76 (29-168) |  | 77 (40-142) |  | 14 (3-29) |  | 6 (2-18) |  | 45 (17-88) |  | 21 (12-37) |  |

^a^median value (25^th^-75^th^ percentiles).

^b^ differences were examined with the Kruskal-Wallis test.

^c^ 5 missing values.

*P* values in bold (*P* < 0.20) correspond to variables considered in the multivariate analysis.
