# Supplementary material for: Association of IL-4 and IL-10 maternal haplotypes with immune responses to P. falciparum in mothers and newborns
Source: BMC Infect Dis. 2013 May 13;13:215. doi: 10.1186/1471-2334-13-215 (PMC3679728; doi:10.1186/1471-2334-13-215)
Supplement: Additional file 4 — Association between maternal cytokine gene polymorphisms and newborns’ antibody levels to P. falciparum antigens (n = 576): univariate analysis. This table summarizes the results of the univariate analysis performed for examining differences between maternal cytokine genotypes or haplotypes and newborns’ IgG levels to 7 recombinant proteins of P. falciparum asexual stage antigens as well as IgM levels to 1 of these antigens. [file 1471-2334-13-215-S4.doc]

**Additional file 4:** Association between maternal cytokine gene polymorphisms and newborns’ antibody levels to *P. falciparum* antigens (n = 576): univariate analysis

| *Maternal cytokine gene (n)* | | *IgG (µg/ml) ^a^* | | | | | | | | | | | | | | *IgM (µg/ml) ^a^* | |
| --- | --- | --- | --- | --- | --- | --- | --- | --- | --- | --- | --- | --- | --- | --- | --- | --- | --- |
|  |  | *AMA-1* | *P ^b^* | *MSP-1* | *P* | *MSP-2/3D7* | *P* | *MSP-2/FC27* | *P* | *MSP-3* | *P* | *GLURP-R0* | *P* | *GLURP-R2* | *P* | *AMA-1 ^c^* | *P* |
| *IL-4_-590_* genotypes: | |  |  |  |  |  |  |  |  |  |  |  |  |  |  |  |  |
|  | *CC* (25) | 618 (247-1279) |  | 23 (3-47) |  | 40 (17-104) |  | 81 (37-135) |  | 9 (2-24) |  | 2 (1-5) |  | 18 (8-25) |  | 0 (0-0) |  |
|  | *CT* (193) | 637 (277-1377) | 0.69 | 32 (6-106) | 0.26 | 58 (25-125) | 0.29 | 62 (28-112) | **0.06** | 5 (1-17) | 0.63 | 3 (1-14) | 0.53 | 27 (9-52) | 0.47 | 0 (0-0) | 0.50 |
|  | *TT* (358) | 603 (215-1238) |  | 26 (6-88) |  | 49 (19-109) |  | 51 (23-104) |  | 5 (2-19) |  | 3 (1-9) |  | 23 (9-50) |  | 0 (0-0) |  |
| *IL-4_+33_* genotypes: | |  |  |  |  |  |  |  |  |  |  |  |  |  |  |  |  |
|  | *CC* (145) | 618 (212-1281) |  | 27 (4-81) |  | 60 (22-130) |  | 54 (26-116) |  | 7 (2-20) |  | 3 (1-11) |  | 24 (11-52) |  | 0 (0-0) |  |
|  | *CT* (290) | 662 (276-1394) | 0.37 | 32 (6-106) | 0.35 | 51 (19-98) | 0.60 | 54 (25-107) | 0.99 | 5 (2-20) | 0.75 | 3 (1-10) | 0.74 | 24 (9-49) | 0.62 | 0 (0-0) | 0.56 |
|  | *TT* (141) | 551 (201-1061) |  | 24 (5-86) |  | 52 (23-124) |  | 53 (24-113) |  | 5 (1-17) |  | 3 (1-9) |  | 23 (9-50) |  | 0 (0-0) |  |
| *IL-4_-590_/IL-4_+33_* haplotypes: | |  |  |  |  |  |  |  |  |  |  |  |  |  |  |  |  |
|  | *No IL4-TT* (145) | 618 (212-1281) |  | 27 (4-81) |  | 60 (22-130) |  | 54 (26-116) |  | 7 (2-20) |  | 3 (1-11) |  | 24 (11-52) |  | 0 (0-0) |  |
|  | *1 copy IL4-TT* (291) | 652 (276-1388) | 0.44 | 32 (6-105) | 0.34 | 51 (19-98) | 0.64 | 55 (25-107) | 0.99 | 5 (2-20) | 0.67 | 3 (1-11) | 0.66 | 24 (9-49) | 0.61 | 0 (0-0) | 0.54 |
|  | *2 copies IL4-TT* (140) | 556 (204-1065) |  | 22 (5-86) |  | 52 (23-125) |  | 53 (24-113) |  | 5 (1-16) |  | 2 (1-9) |  | 23 (9-50) |  | 0 (0-0) |  |
| *IL-10_-1082_* genotypes: | |  |  |  |  |  |  |  |  |  |  |  |  |  |  |  |  |
|  | *GG* (47) | 585 (235-1280) |  | 29 (6-110) |  | 68 (33-167) |  | 63 (34-138) |  | 6 (3-22) |  | 2 (1-7) |  | 27 (10-47) |  | 0 (0-0) |  |
|  | *GA* (223) | 546 (203-1024) | **0.13** | 27 (4-84) | 0.42 | 43 (16-98) | **0.02** | 51 (21-101) | **0.16** | 5 (2-16) | **0.12** | 3 (1-10) | 0.87 | 25 (9-50) | 0.82 | 0 (0-0) | 0.57 |
|  | *AA* (306) | 714 (287-1412) |  | 28 (6-105) |  | 56 (21-125) |  | 54 (27-115) |  | 5 (2-22) |  | 3 (1-12) |  | 22 (9-51) |  | 0 (0-0) |  |
| *IL-10_-819_* genotypes: | |  |  |  |  |  |  |  |  |  |  |  |  |  |  |  |  |
|  | *CC* (191) | 591 (209-1220) |  | 28 (4-94) |  | 54 (24-114) |  | 56 (25-112) |  | 5 (1-16) |  | 3 (1-8) |  | 24 (8-50) |  | 0 (0-0) |  |
|  | *CT* (293) | 630 (239-1397) | 0.50 | 29 (6-86) | 0.89 | 51 (20-110) | 0.51 | 53 (23-106) | 0.44 | 7 (2-20) | 0.64 | 4 (1-13) | **0.07** | 24 (9-50) | 0.89 | 0 (0-0) | 0.92 |
|  | *TT* (92) | 769 (317-1216) |  | 21 (4-128) |  | 41 (17-122) |  | 55 (30-112) |  | 5 (2-23) |  | 2 (1-6) |  | 20 (10-53) |  | 0 (0-0) |  |
| *IL-10_-592_* genotypes: | |  |  |  |  |  |  |  |  |  |  |  |  |  |  |  |  |
|  | *CC* (191) | 591 (212-1220) |  | 29 (5-98) |  | 54 (24-114) |  | 56 (25-111) |  | 5 (2-17) |  | 3 (1-8) |  | 25 (9-50) |  | 0 (0-0) |  |
|  | *CA* (294) | 633 (241-1403) | 0.61 | 29 (6-86) | 0.89 | 50 (20-110) | 0.60 | 51 (22-105) | **0.16** | 7 (2-20) | 0.60 | 4 (1-13) | **0.08** | 24 (9-50) | 0.92 | 0 (0-0) | 0.97 |
|  | *AA* (91) | 737 (313-1115) |  | 20 (4-108) |  | 44 (17-124) |  | 63 (32-118) |  | 4 (1-20) |  | 2 (1-6) |  | 19 (10-53) |  | 0 (0-0) |  |
| *IL-10_-1082_/IL-10_-819_/IL-10_-592_* haplotypes: | |  |  |  |  |  |  |  |  |  |  |  |  |  |  |  |  |
|  | *No IL10-ATA* (196) | 591 (210-1205) |  | 29 (5-98) |  | 54 (23-117) |  | 57 (25-112) |  | 5 (1-16) |  | 3 (1-8) |  | 25 (9-50) |  | 0 (0-0) |  |
|  | *1 copy IL10-ATA* (293) | 630 (246-1397) | 0.54 | 29 (6-86) | 0.92 | 51 (20-108) | 0.54 | 51 (22-105) | **0.19** | 7 (2-21) | 0.48 | 4 (1-13) | **0.09** | 24 (9-50) | 0.83 | 0 (0-0) | 0.89 |
|  | *2 copies IL10-ATA* (87) | 762 (313-1218) |  | 20 (5-108) |  | 42 (17-124) |  | 63 (31-118) |  | 4 (2-20) |  | 2 (1-6) |  | 18 (10-50) |  | 0 (0-0) |  |
| IL-13_-1055_ genotypes: | |  |  |  |  |  |  |  |  |  |  |  |  |  |  |  |  |
|  | *CC* (186) | 663 (235-1396) |  | 22 (4-109) |  | 53 (20-131) |  | 63 (27-114) |  | 5 (2-17) |  | 3 (1-13) |  | 27 (9-55) |  | 0 (0-0) |  |
|  | *CT* (295) | 652 (281-1331) | **0.12** | 30 (6-86) | 0.71 | 53 (22-112) | **0.18** | 52 (25-116) | 0.42 | 5 (1-19) | 0.99 | 2 (1-8) | **0.10** | 21 (9-48) | 0.28 | 0 (0-0) | 0.90 |
|  | *TT* (95) | 520 (192-925) |  | 27 (6-86) |  | 44 (21-79) |  | 51 (27-92) |  | 6 (2-17) |  | 4 (1-12) |  | 24 (11-51) |  | 0 (0-0) |  |

^a^median value (25^th^-75^th^ percentiles).

^b^ differences were examined with the Kruskal-Wallis test.

^c^ 1 missing value.

*P* values in bold (*P* < 0.20) correspond to variables considered in the multivariate analysis.
